# IgE versus IgG and IgA: Differential roles of allergen‐specific antibodies in sensitization, tolerization, and treatment of allergies

**DOI:** 10.1111/imr.13386

**Published:** 2024-09-16

**Authors:** E. F. Knol, R. J. J. van Neerven

**Affiliations:** ^1^ Department of Dermatology/Allergology UMC Utrecht Utrecht the Netherlands; ^2^ Cell Biology and Immunology Wageningen University & Research Wageningen the Netherlands

**Keywords:** allergy, blocking antibody, immunological mechanism, immunotherapy, sensitization, tolerance

## Abstract

The prevalence of asthma, rhinitis, and food allergies has increased dramatically over the last few decades. This increase originally started in western countries, but is now also evident in many other regions of the world. Given the fact that the increase is so quick, the noted increase cannot be linked to a genetic effect, and many environmental factors have been identified that are associated with increased or reduced prevalence of allergies, like changing dietary habits, increased urbanization, pollution, exposure to microorganisms and LPS, and the farming environment and raw milk consumption. Although the key role of allergen‐specific IgE in allergies is well known, the role of allergen‐specific IgG and IgA antibodies is less well defined. This review will provide an overview of the functions of allergen‐specific IgE in allergy, the role of allergen‐specific antibodies (IgG (4) and IgA) in allergen immunotherapy (AIT), the possibility to use allergen‐specific antibodies for treatment of ongoing allergies, and the potential role of allergen‐specific antibodies in tolerance induction to allergens in a preventive setting. In the last, more speculative, section we will present novel hypotheses on the potential role of allergen‐specific non‐IgE antibodies in allergies by directing antigen presentation, Th2 development, and innate immune training.

## 1 INTRODUCTION

**FIGURE 1:**
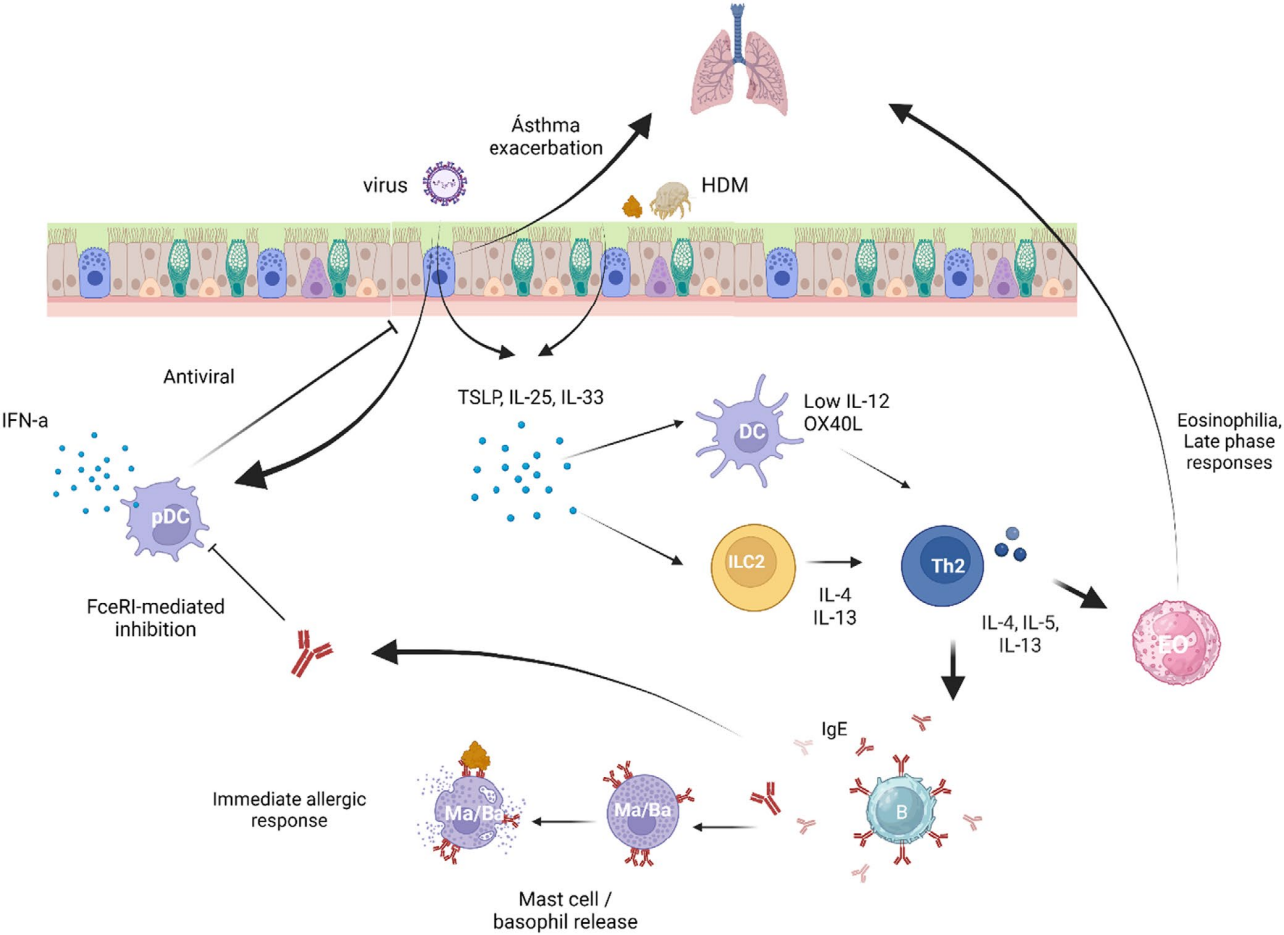
Allergic sensitization and effector mechanisms in allergy. The alarmins IL‐33, IL‐25, and TSLP induce differentiation of ILC2 and Th2 cells. The IL‐4 and IL‐13 they produce induce B cell class switching to the production of IgE, and IL‐5 supports eosinophil survival. IgE binds to high affinity IgE receptors (FcεRI) on basophils and mast cells. These cells release histamine and other proinflammatory mediators into the surrounding tissue upon crosslinking by binding to an allergen. IgE can also trigger FcεRI on pDC, which leads to a reduction in antiviral IFN‐α production.

The prevalence of IgE‐mediated allergies such as allergic asthma, rhinitis, and food allergies has increased dramatically over the last few decades. IgE mediated allergies are dependent on the formation of allergen‐specific IgE during the sensitization phase (Figure 1). In 1990 it was shown that in human allergic patients, allergen‐specific Th2 cells exist that support IgE production by B cells.^1^

After crossing the epithelial barrier, allergens are taken up by dendritic cells, processed and presented to naïve T cells that differentiate into Th2 cells. In both asthma and food allergies alarmins are produced by the epithelium after exposure to allergens. These alarmins such as IL‐33, IL‐25, and TSLP play an important role in promoting ILC2 and Th2 responses either directly or via dendritic cells.^2^, ^3^, ^4^

The IL‐4 and IL‐13 produced by these Th2 and ILC2 cells induce IgE class switching in B cells, resulting in the production and circulation of allergen‐specific IgE. After sensitization, allergen‐specific IgE circulates in the blood and binds to the high affinity IgE receptor (FcεRI) on mast cells and basophils. After re‐exposure to allergens, these cells release a range of proinflammatory mediators including histamine, that lead to immediate type allergic responses. Depending on host genetic factors, the route and dose of allergen exposure, these allergic reactions can occur in different tissues and lead to different types of clinical allergies such as allergic asthma, rhinitis, food allergy, atopic dermatitis, but also drug allergy and insect bite hypersensitivity.

Although the key role of allergen‐specific IgE in allergies is well known, the role of allergen‐specific IgG and IgA antibodies is less well defined. Allergen‐specific antibodies play a role in tolerization, sensitization, allergic symptoms, and are induced or administered in specific treatment of allergies. Allergen‐specific IgG and IgA antibodies are also present in our diet where they potentially play a role in tolerization. Allergen‐specific IgE antibodies are produced after sensitization, and cause allergic symptoms—but also increase allergen uptake across epithelial barriers. Allergen‐specific IgG (4) and IgA antibodies are mainly induced by allergen immunotherapy, block functional IgE responses, and are even used as therapeutic agents. Recent findings have suggested that allergen‐specific IgG (4) may antagonize systemic responses to allergens, and allergen‐specific IgA may antagonize responses to allergens on mucosal surfaces.

The current review will not discuss the immunological functions of the cells of the immune system in allergy in detail, but will rather focus on the role of the allergen‐specific antibodies of the IgE, IgA, and IgG type. These antibodies have a function in allergic disease, but can also play role in the prevention of allergies, explain part of the clinical effects of allergen immunotherapy, and can even be used as therapeutic agents. In addition, several new developments led us to speculate on novel functions of allergen‐specific antibodies that are emerging.

## 2 THE FUNCTIONAL EFFECTS OF ALLERGEN‐SPECIFIC IgE IN ALLERGY

### 2.1 History of IgE

IgE is the immunoglobulin isotype in humans with the lowest concentration in the blood. It was not until the late 60s of the previous century that IgE was discovered in parallel by two independent groups from USA and Sweden.^5^, ^6^ The half‐life of IgE in serum is very low, due to the rapid binding via its Fc‐part to its high affinity receptor FcεRI. This receptor has a [Kd] of approximately 1 × 10‐10 M, and is mostly found on basophils and mast cells. In contrast to most other isotypes, the IgE molecule contains four heavy chain C regions (Cε1–Cε4). The concentration of IgE in serum of healthy controls is below 240 ng/mL, compared to approximately 15 mg/mL for IgG. An important aspect of IgE is that due to its low serum levels, the proportion of IgE that is specific to allergens is relatively high. This is relevant because with a high prevalence of specific IgE antibodies, allergens bind to the IgE on mast cells or basophils and can crosslink the FcεRI. If the crosslinking is of sufficient strength^7^ activation and degranulation of these cells is induced, resulting in release of pre‐stored and newly synthesized inflammatory mediators and cytokines, such as histamine, leukotrienes and interleukin‐4 (IL‐4).

### 2.2 IgE class switching

IgE producing B cells are rare in the periphery and our knowledge in humans has been rather limited as compared to murine models.^8^ By transcriptome analysis of the allergen specific IgE producing B cells in humans it was demonstrated that these were primarily plasmablasts, in contrast to allergen‐specific IgG clones that were memory or naive B cells.^9^ These memory B cells were marked by expression of high levels of the low affinity IgE receptor CD23 and were MHC Class II^high^, indicating a more immature phenotype of plasmablast. Remarkably, low levels of Syk suggested a reduced survival ability of these IgE producing plasmablasts. For peanut‐specific IgE this was further confirmed demonstrating that these IgE producing plasmablasts are not memory B cells.^9^

The cytokine IL‐4 induces IgE class‐switching in naive B cells. This IL‐4 can be derived from type 2 T helper cells (Th2) or basophils, but it is most probably IL‐4 derived from the population of T follicular helper (Tfh) cells that is required to initiate class‐switch due to their location in the B cell follicle where they can directly interact.^10^ Another Th2 cytokine, IL‐13, can promote this process, while the costimulatory CD40/CD40L signal is required to initiate this class‐switching in naive B cells.^11^

During the class switching, the variable, diversity, and joining regions gene segments—that have rearranged previously to determine the antibody specificity, and that are most proximal to the gene of the μ constant region, are linked to the ε‐constant region by excision of the intermediate DNA. After transcription, this resulting in mRNA that encodes IgE with the same specificity as the parental IgM‐producing B cell.

### 2.3 Binding of IgE to FcεRI on basophils and mast cells and mediator release

The binding of IgE to the FcεRI on mast cells and basophils is by the Cε3–Cε4 heavy chain part of IgE and is very stable due to the high affinity binding of the receptor. It has been shown that IgE binding to the FcεRI on mast cells is prolonging its survival.^12^ The FcεRI expression level is positively related to the IgE concentration.^13^ When allergens bind to IgE and crosslink the FcεRI the signaling events for cell activation requires specific properties of these interactions. First, there need to be about 100–1000 crosslinks per single cell for at least 100 s. Second, the minimal distance between cross‐linked IgE molecules is estimated to be 8–10 nm and the maximal distance to make a stable contact is estimated to be 20–24 nm.^7^ When these requirements are fulfilled the cross‐linking of the FcεRI initiates a chain of phosphate transfers within the receptor microenvironment. The mechanism by which the IgE binding α‐chain aggregation is sensed by the signaling subunits is still unknown. Lyn can be constitutively associated with FcεRI, but is incapable of phosphorylating the receptor subunits in the absence of aggregation. One of the first signals is the activation of β‐chain bound Lyn.^14^ The model of transphosphorylation by Metzger and coworkers^15^ proposes that Lyn phosphorylates an adjacent FcεRI following aggregation of at least two receptors. In contrast, in a model by Baird and coworkers, it is stated that Lyn is constitutively active in so‐called lipid rafts and that cross‐linking of FcεRI promotes its proximity to active Lyn in these lipid rafts.^16^ Subsequently, Lyn phosphorylates the β and γ‐chain immunoreceptor tyrosine activation motifs (ITAMs). The phosphorylated γ‐chains then recruit the PTK‐Syk, which upon binding to the two phosphorylated ITAMs tyrosyls also undergoes activation.^17^ This mechanism illustrates the requirement that clustered FcεRIs remain in close proximity for at least the time required for these processes to take place. The activated Syk subsequently tyrosine phosphorylates phospholipase C‐γ1 and phospholipase C‐γ2. These phosphorylated phospholipase Cγs catalyze the hydrolysis of plasma membrane phosphatidylinositol 4,5‐bisphosphate, generating inositol 1,4,5‐trisphosphate and 1,2‐diacylglycerol. These second messengers promote release of Ca^2+^ from internal stores and activate PKC, respectively. Both events are essential for FcεRI ‐mediated degranulation.^18^

### 2.4 IgE‐mediated allergen presentation to Th2 cells

**FIGURE 2:**
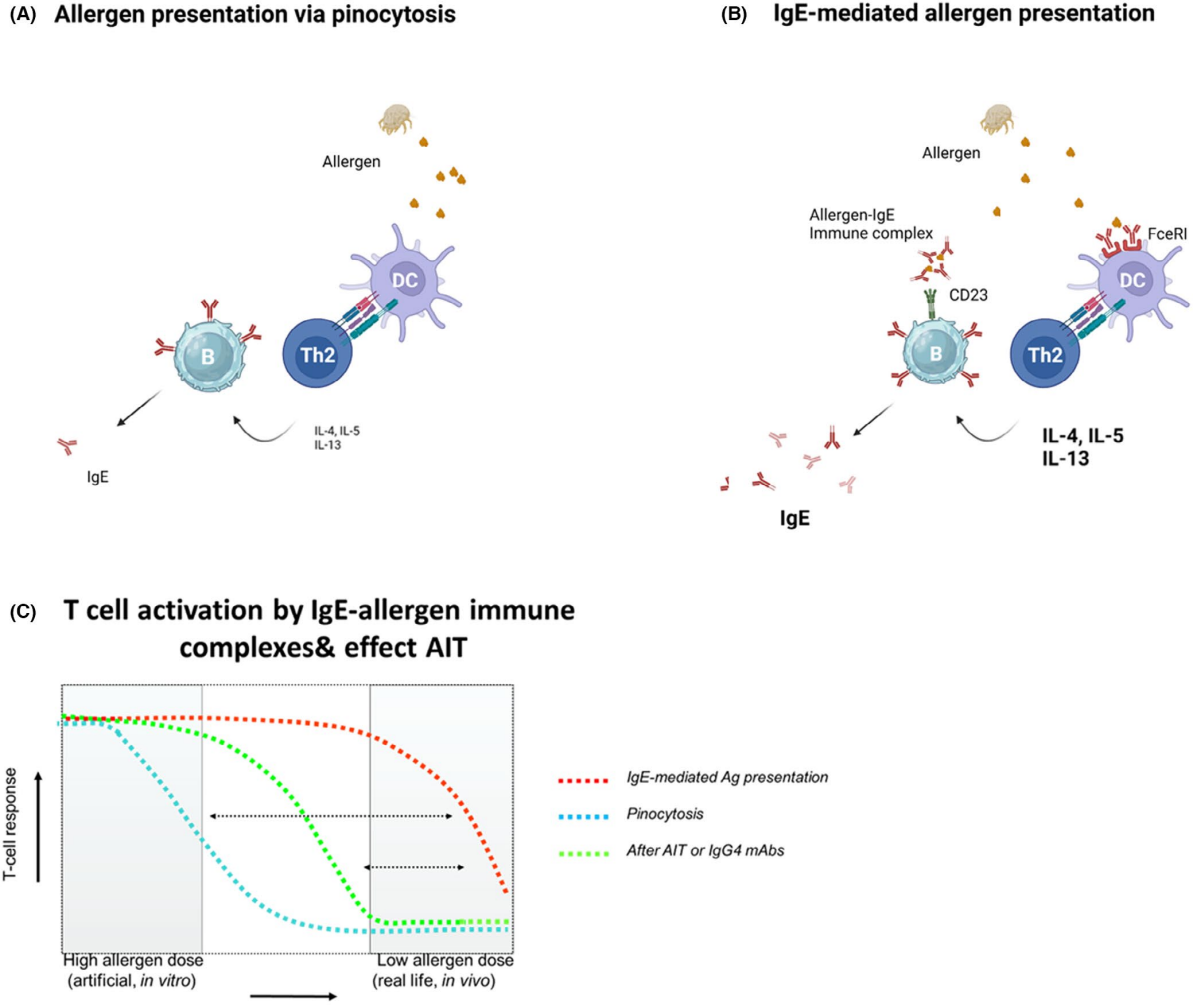
Normal antigen presentation of allergens occurs by uptake of proteins via pinocytosis. (A) This process is not very efficient and works best at high allergen exposure levels. In contrast, IgE‐mediated allergen presentation via Fcε receptors CD23 and FcεRI is highly efficient and requires lower allergen levels, representing in vivo exposure levels of aeroallergens or trace amounts for food allergens. (B) The difference in allergen dose needed for both processes is a factor of around 100. Immunotherapy and anti‐IgE have been shown to reduce the efficiency of IgE‐mediated allergen presentation considerably (C).

In addition to the effects of IgE on mast cells and basophils, it is well established that IgE also plays an important role in the continuous activation of pre‐existing allergen‐specific Th2 cells. IgE can capture allergens and increase allergen presentation of allergens to specific T cells via binding to the low affinity IgE receptor CD23—mainly expressed on B cells—and via the high affinity IgE receptor that can be expressed on monocytes and dendritic cells as well (reviewed in^19^). This process of IgE‐mediated allergen presentation is receptor mediated and is 10–100 fold more efficient than normal, pinocytosis‐dependent antigen presentation.^20^, ^21^, ^22^ As a result this means that at low allergen exposures, like in respiratory allergies, allergen specific T cells can still be activated efficiently (Figure 2).

Interestingly, two recent publications have demonstrated that in allergic patients type‐2 committed memory B cells exist that are IgG1+ (or IgG4) and IL4R^hi^ and CD23^hi^.^23^, ^24^ These cells are poised to switch to the production of IgE. As IgE producing B cells are rather short lived, these pre‐committed memory B cells are a pool of B cells that enable long term IgE memory. Notably, their high expression of CD23 may indicate that IgE‐mediated allergen presentation via B cells targets these pre‐committed B cells when presenting allergens to pre‐existing Th2 cells—which typically happens at low allergen exposure levels—will switch to the production of IgE, and are involved in the long term IgE memory formation in allergies.

Allergen‐specific B cells from birch and peanut allergic patients were enriched in these pre‐committed allergen‐specific B cells, indicating that in IgE‐mediated allergen presentation via CD23 especially allergen‐specific B cells will be involved in allergen presentation to Th2 cells.^24^ It has been known since the 1980s that specific B cells are more efficient antigen presenting B cells than non‐specific B cells.^25^

However, when allergens are already bound to antibodies (neutralized by IgG or bound by IgE), allergen‐specific B cells may primarily need to use Fc receptor mediated antigen presentation as the B cell epitopes of the allergens are not available to the surface Ig of the specific B cells.

Interestingly, IgE‐mediated allergen rpesentation can be blocked by allergen immunotherapy (AIT),^26^, ^27^, ^28^, ^29^ as well as by anti‐IgE antibodies.^30^ Since these early findings, the IgE‐facilitated allergen binding (IgE‐FAB) and ELIFAB assays has been developed as a proxy for IgE‐mediated T cell antigen presentation.^31^, ^32^ The inhibition of IgE‐mediated allergen presentation after AIT is the result of the induction of high levels of allergen‐specific IgG (4) antibodies, that will be discussed in more detail later.

### 2.5 IgE inhibits IFN‐α production in pDC

Another remarkable role of IgE in allergic asthma is that it antagonizes the production of the antiviral cytokine IFN‐α in plasmacytoid DC, thus increasing the susceptibility to viral respiratory infections, that I turn can exacerbate asthma.

Triggering of FcεRI on pDCs by crosslinking anti‐IgE antibodies was shown to inhibit the production of IFN‐α after TLR‐7/8 activation by respiratory ssRNA viruses or TLR ligands.^33^, ^34^ In contrast to the inhibitory action of IgE on pDCs, IgG‐containing immune complexes have been shown to promote IFN‐α production in pDC,^35^ and CD32‐dependent increases in IFN‐α production have been noted as well.^36^ This suggests that where FcεRI triggering inhibits IFN‐α production in pDC, IgG‐containing immune complexes actually promote IFN‐α production by interacting with CD32, the low affinity IgG receptor.

This idea is supported by the fact that anti‐IgE treatment in asthma is associated with increased antiviral immunity in asthma patients. Anti‐IgE treatment led to restoration of IFN‐α production in pDC after TLR triggering.^37^, ^38^ In addition, recent studies have shown a reduction of viral respiratory infections after AIT.^39^, ^40^ AIT also leads to an increase in pDC^41^ as well as increased IFN‐α production after stimulation with the TLR9 ligand CPG.^42^

A possible explanation of the data from Woehlk et al. is that allergen‐specific IgG (administered passively or AIT‐induced) may prevent FcεRI‐mediated inhibition of IFN‐α inhibition in pDC either by neutralizing the allergen, or by actively engaging CD32 when the IgG is complexes with allergens. If this is indeed the case, the role of allergen‐specific IgG antibodies induced by AIT may also play a role in reducing the susceptibility of respiratory infections in asthma patients.

### 2.6 Anti‐IgE targeted therapies

Because of its key role in type 1 allergy, anti‐IgE antibodies have been developed and approved for treatment of allergic asthma.^43^, ^44^ Free serum IgE levels decrease sharply after anti‐IgE treatment, as does FcεRI expression on basophils^45^ and dendritic cells.^46^ In many allergic diseases blocking IgE with the anti‐IgE antibody Omalizumab has beneficial effects. Initially, its effect was demonstrated in moderate‐to‐severe allergic asthma and in patients with intermittent (seasonal) and persistent (perennial) allergic rhinitis. In addition, also in other diseases such as urticaria removal of IgE by omalizumab results in rapid significant clinical improvement.^47^ Although originally more than 20 years ago it was indicated that anti‐IgE improved severity in peanut allergy^48^ only recently was this substantiated for several food allergies and is now FDA approved (https://www.fda.gov/news‐events/press‐announcements/fda‐approves‐first‐medication‐help‐reduce‐allergic‐reactions‐multiple‐foods‐after‐accidental) for the treatment of food allergies.^49^ These data underline the key role of IgE in allergic diseases.

## 3 ORAL DELIVERY OF ALLERGEN‐SPECIFIC IgG AND IgA ANTIBODIES: BREASTMILK AND COW'S MILK

In early life infants are not only exposed to allergens, but also receive allergen‐specific antibodies orally through breastfeeding and consumption of (unprocessed or pasteurized) cow's milk.

The presence of food allergens in breast milk has been known for a long time, and the presence of inhalation allergens such as house dust mite and pollen allergens has been demonstrated as well, leading to discussion on their protective or promoting role in sensitization.^50^, ^51^, ^52^, ^53^, ^54^, ^55^, ^56^, ^57^ In addition to allergens, breast milk also contains allergen‐specific IgA and IgG antibodies. The presence of allergen‐specific IgA in breastmilk has been shown to contribute to protection against cow's milk allergy.^58^, ^59^, ^60^ Likewise, the total levels of IgA in breast milk are inversely associated with AD in early life,^61^ suggesting a role for allergen‐specific antibodies in breastmilk in promoting tolerance to allergens.

**FIGURE 3:**
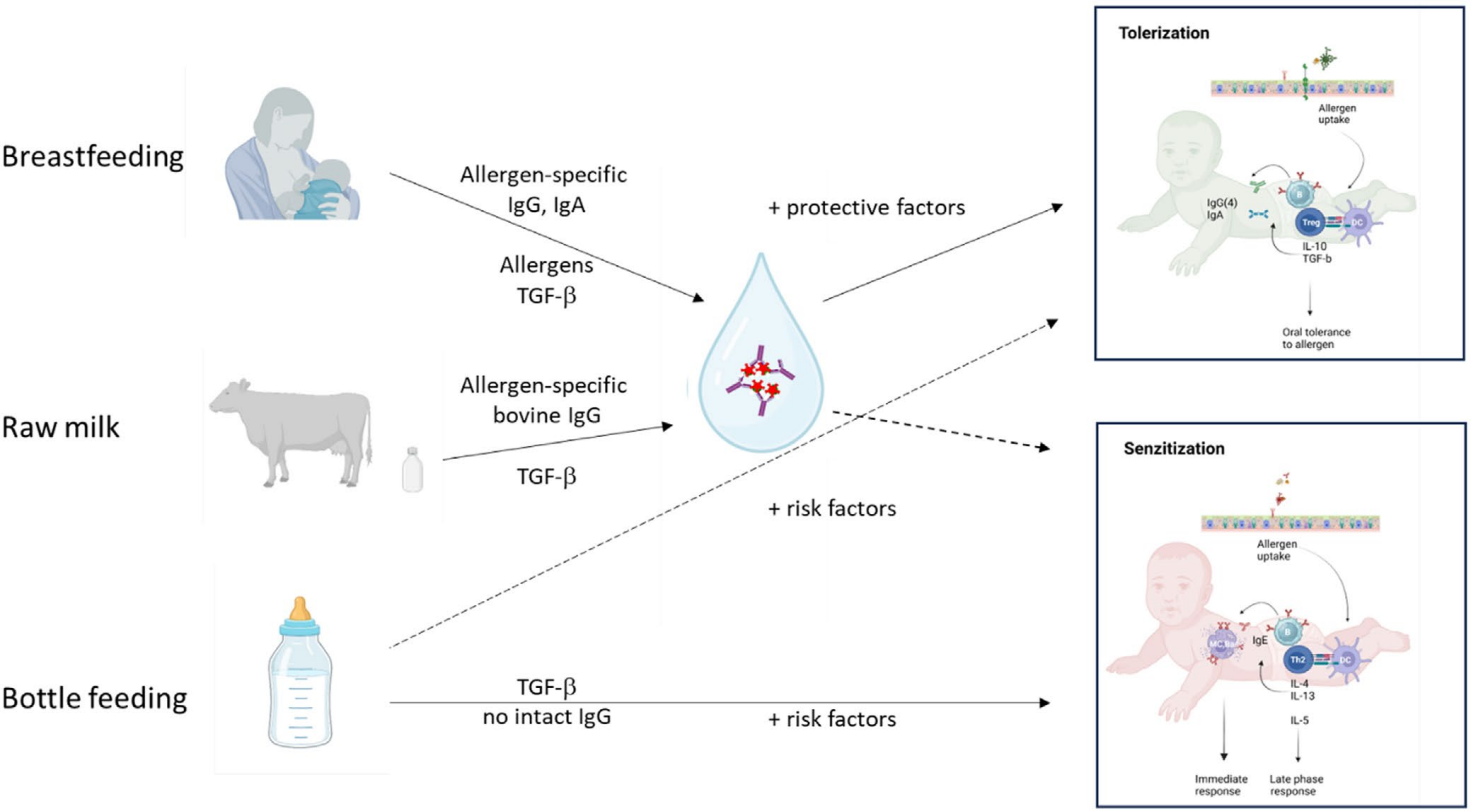
A putative role of (breast) milk‐derived allergen‐specific IgG in the prevention of allergies. Breastmilk and raw and pasteurized cow's milk contain allergen‐specific IgG (and IgA) antibodies, and the presence of allergens and allergen‐IgG immune complexes has been shown in breastmilk. Such complexes may also form locally in the oro‐ or nasopharynx after swallowing of nasal secretions that contain inhaled allergens, as well as swallowing of raw/pasteurized milk. In almost all current infant feeding products and powdered milk, however, IgG is not present as functional protein. As allergen‐IgG immune complexes induce tolerance before sensitization in animal models, these potentially have the same effect in humans., and may in part explain why raw milk consumption is associated with fewer respiratory allergies.

Evidence is emerging from several animal models that allergen‐immune complexes may play a role in such protection. Allergen‐IgA immune complexes have been shown to induce oral tolerance in neoanatal mice in an animal model.^62^ In addition to IgA‐allergen immune complexes, also allergen‐IgG immune complexes have been demonstrated to promote immune regulation and tolerance induction (Figure 3). Such immune complexes have been detected in human cord blood^63^ as well as in human milk.^64^, ^65^ Allergen‐IgG immune complexes have been shown to promote regulatory T cell responses and contribute to allergy prevention in food allergy and asthma models.^65^, ^66^, ^67^, ^68^

Another source of allergen‐specific IgG and IgA antibodies is unprocessed or pasteurized cow's milk. Consumption of unprocessed milk is related to lower asthma and hay fever incidence (reviewed in^69^, ^70^, ^71^). Several milk proteins, including bovine IgG have been suggested to play a role in this effect.^72^ Bovine milk‐derived IgG (bIgG) is known to bind to many different bacteria, but cow's milk also contains high RSV‐specific IgG levels, which can neutralize RSV infection in vitro and experimental infection in vivo. bIgG binds to human Fc receptors, promotes phagocytosis and can promote the proliferation of RSV‐specific T cells.^73^, ^74^, ^75^ Interestingly, cow's milk has been reported to also recognizes allergens such as mites, fungi, pollen, and wheat.^76^ In a more recent paper, we have in a more extensive analysis been able to show that although cow's milk contains IgG to grass and tree pollen allergens, and only low levels to cat and dog allergens, the levels of IgG to fungi and house dust mite were consistently high (*Kno*l et al. *unpublished data*).

As allergens are swallowed after deposition in the nasal cavity, they can come into contact with orally delivered breast milk or cow's milk.^77^ This can theoretically lead to the formation of IgG‐allergen immune complexes that come into contact with the immune tissue in the Waldeyer's ring surrounding the naso‐ and oropharynx (tonsils, adenoids) before reaching the acidic environment of the stomach and the proteolytic enzymes in the small intestine.

Such a route could explain how allergen‐specific antibodies, either present in milk as immune complex, or upon binding to allergens in the oropharynx or nasopharynx can play a role in the protection against the sensitization to allergens. If cow's milk allergen‐specific antibodies can indeed promote tolerance and prevent sensitization is not known at present though.

As there may be a potential role of immune complexes in prevention of allergies, maternal vaccination against (especially food?) allergens may be considered as a relevant route to help prevent sensitization in allergies in addition to optimized timing of introduction of novel foods.

## 4 THE ROLE OF IgG AND IgA ALLERGEN‐SPECIFIC ANTIBODIES IN ALLERGY

Already perinatally non‐IgE antibodies specific for allergens are present in fetal and newborn blood and the gastrointestinal tract, in part via passive transfer of maternal IgG in utero, or consumption of IgG and IgA via breastmilk. These other isotypes in competition with allergen‐specific IgE can affect the clinical responses after allergen exposure. High levels of cat and birch‐specific IgG antibodies in cord blood are associated with less allergies in the first eight years of life^78^ and low levels of allergen‐specific IgA in predispose for cow's milk allergy.^59^ The most likely explanation is that these other isotypes can block the access of allergens to its specific IgE bound on mast cells and basophils.^79^ In vitro models have shown that increasing allergen doses can overcome the blocking effect of the other isotypes, as demonstrated in basophil activation testing.^80^ Other explanations have been the simultaneous interaction of IgG with inhibitory CD32b IgG receptors and IgE with FcεRI on the mast cell, leading to inhibition of mast cell degranulation.^81^, ^82^ However, this is mostly described in artificial models, but in the human in vivo effects has some constraints.^83^, ^84^ Another indirect explanation can be that IgG4, as well as IgA, production is promoted by the immunoregulatory cytokines IL‐10 and TGF‐β and it is this IL‐10 and or TGFβ that are directly inhibiting basophil and mast cells activation, including inhibiting FcεRI expression, cytokine production and degranulation.^85^, ^86^, ^87^, ^88^ There is some inconsistency between different studies on the relation between these isotypes The relation between ratios of allergen‐specific IgE and the other isotypes depends on the type of allergen.

It has been shown that in cow's milk, peanut and birch pollen allergic patients allergen specific IgE is in the same order of magnitude, but that there is a large difference up to 100‐fold, in the concentrations of allergen‐specific IgG1 and IgG4 between these patient groups.^89^ Binding of immune complexes that consisted of allergens incubated with the patients' serum to B lymphocytes showed that this immune complex binding was via CD23 and complement receptors, not IgG receptors, on B cells. Whereas IgG4 are unique in the lack of complement activating properties when compared to the other IgG isotypes, might this implicate that allergen‐specific IgG4 is very efficient in neutralizing allergen‐IgE interactions both on mast cells/basophils and on B cells.

## 5 IMMUNOLOGICAL EFFECTS OF ALLERGEN IMMUNOTHERAPY (AIT): THE ROLE OF ALLERGEN‐SPECIFIC IgG AND IgA ANTIBODIES

Although many pharmacological treatments are available, and several biologicals have reached the clinic over the past two decades, to date AIT is the only effective, causal treatment for IgE‐mediated allergies such as allergic rhinitis that has sustained, long term efficacy.^90^, ^91^, ^92^, ^93^ AIT has been used for over a century after its introduction by Noon in 1911.^94^ It is an effective treatment based on the exposure with the allergen one is allergic to. AIT is very effective in the treatment of allergic asthma, hay fever and bee venom allergies, and oral immunotherapy strategies for food allergy are currently under development.

In addition to the classical subcutaneous immunotherapy many different administration routes (and adjuvants) are in use today, ranging from subcutaneous, epicutaneous, sublingual (spit/swallow), oral tablet (inhalation allergens), oral (foods), to intralymphatic administration of allergens, as well as passive AIT with monoclonal allergen‐specific IgG4 antibodies (see next section). In addition, combination treatment of AIT with anti‐IgE and with passive AIT has been performed. These different routes and approaches are not the topic of this review, and have been discussed in detail elsewhere.^95^, ^96^, ^97^

AIT redirects the allergic immune response form a Th2‐dominant response to a more regulatory immune response.^98^, ^99^, ^100^ This leads to a clinical improvement and a reduced influx of eosinophils and CD4+ Th2 into target tissues, in combination with reduced production of Th2 cytokines.^101^, ^102^ In addition, AIT leads to the production of IL‐10 by monocytes and T cells, and induces regulatory T cells (Treg).^98^, ^103^, ^104^ In addition to its immunoregulatory function, IL‐10 is instrumental in inducing class switching to IgG4 antibodies specific to the allergen.^105^, ^106^

In more recent studies the induction of IL‐10 producing regulatory B cells (Bregs) have been demonstrated after AIT.^107^, ^108^ In addition, instead of “classical” ILC2, after AIT IL‐10+ ILC2 are induced, that have a more tolerogenic phenotype.^109^ Two other studies have shown a reduction in ILC2 after AIT.

In the first paper, Eljaszewics demonstrated a change in peripheral blood phenotypes after AIT, with a reduction in ILC2 and a shift from pro‐inflammatory to anti‐inflammatory monocytes.^41^ Likewise, a durable decrease in ILC2 and increased ILC1 and ILC3s have been reported after AIT.^110^ These studies underline the shift from a type 2 dominated immune response to allergens to a more regulatory type of immune response.

These changes in immune responses are also reflected in the types of allergen‐specific antibodies that are produced. After an initial, transient, increase in allergen‐specific IgE antibodies high levels of allergen‐specific IgG, especially IgG4, are induced by AIT, and allergen‐specific IgG4 is still today one of the best recognized biomarkers for immunotherapy^111^ Allergen‐specific IgG4 antibodies play an important role in blocking both the IgE‐mediated release of inflammatory mediators from mast cells and basophils,^112^, ^113^, ^114^ but also the IgE‐facilitated activation of allergen‐specific Th2 cells.^26^, ^27^, ^28^, ^29^, ^91^

Blocking of IgE‐allergen binding was mostly linked to the presence of allergen‐specific total IgG,^26^ IgG4,^115^ but also other IgG isotypes like IgG1^116^ and possibly IgG2.^117^ Serum IgM or IgA after AIT did not inhibit the binding of allergen‐IgE immune complexes to B cells.^26^

However, whereas a recent report demonstrated the inhibition of IgE‐mediated basophil and mast cell degranulation by IgA in a food allergy model,^118^ other studies could not demonstrate an inhibitory role of AIT‐induced allergen‐specific IgA on the binding of IgE to the allergens,^119^ or of binding of IgE‐allergen immune complexes to B cells.^120^

Although the levels of allergen‐specific IgG and IgA antibodies after AIT are increased they do not per se correlate with the clinical efficacy. However, the inhibition of IgE‐allergen complex binding to CD23 on B cells has been shown to correlate better with clinical efficacy than the allergen‐specific IgG4 levels after AIT.^28^, ^31^, ^111^ In addition, also IgG4 from nasal fluid after AIT has been shown to have inhibitory activity in this assay, and correlates with the clinical response as well.^121^

IgA antibodies are the main isotype found in mucosal tissues in the intestines and respiratory tract—and these are also the locations where the allergens are deposited upon exposure. Therefore, a protective role of allergen‐specific IgA antibodies present on mucosal surfaces against the uptake of allergens would make sense. Indeed, after grass pollen injection immunotherapy, allergen‐specific IgA2 and to a lesser extent IgA1 antibodies were shown to be induced in serum.^120^ These IgA antibodies could induce IL‐10 production after crosslinking of IgA coated monocytes, as was also shown by Geissmann et al., that demonstrated that polymeric IgA complexes induced the production of IL‐10 and DC activation via CD89.^122^ In contrast to subcutaneous immunotherapy, after SLIT more higher allergen‐specific IgA1 antibodies are present in serum and nasal fluid compared to allergen‐specific IgG4.^123^

Interestingly, the mucosal introduction of allergen‐IgA immune complexes into the respiratory tract has been shown to be able to prevent sensitization, but also to induce tolerance induction after systemic sensitization in a murine model,^124^ and allergen‐IgA immune complexes have been shown to induce oral tolerance in neonatal mice.^62^ As described above, similar findings were reported for breast milk IgG‐allergen immune complexes.^65^, ^66^, ^68^

These findings indicate that apart from a blocking effect allergen‐specific IgG and IgA antibodies may, when complexed to allergens, also play an active tolerizing role in the prevention of allergies as well as after AIT.

## 6 PASSIVE AIT WITH ALLERGEN‐SPECIFIC IgG4 ANTIBODIES

Even though the blocking activity of allergen‐specific IgG (4) does not explain all immunological effects induced by AIT, it has been proposed that pre‐seasonal treatment of allergic patients with monoclonal IgG4 blocking antibodies (mimicking part of the therapeutic effect of AIT) might be a novel approach for treatment of allergies.^125^ This approach seems most viable for allergies which are mainly linked to a single allergenic protein such as cat allergy (Fel d 1) and birch allergy (Bet v 1).

IgG4 constitutes approximately 2.5% of all serum IgG,^126^ and it has been noted that IgG4 in serum is functionally monovalent and even bispecific.^127^, ^128^ This is the result of Fab arm exchange, that only occurs in IgG4 because of an unstable hinge region.^129^ Because of the absence of bivalent IgG4s, IgG4 cannot bind with two Fab arms to a multivalent allergen, which results in lower avidity and the formation of smaller immune complexes.^128^ It should be noted that when used for passive immunotherapy, the IgG4 antibodies used have stabilized hinge regions to prevent this Fab arm exchange.

Several recent studies have demonstrated that passive administration of human allergen‐specific IgG (4) mAbs can indeed be clinically effective in allergic patients.^130^, ^131^, ^132^, ^133^ More recently, also a panel of Ara h 2‐IgG4 mAbs have been developed that bind to epitopes in Ara h2 and other peanut allergens and that can prevent anaphylaxis,^134^ and has synergistic activity with oral immunotherapy for peanut in mice (Gasser et al. *unpublished data*). Interestingly, injected IgG4 antibodies alone lead to reduced nasal Th2 cytokines in humans, indicating not only an IgE blocking activity, but also a reduced Th2 activation in vivo.^130^

We also noted a more immunoregulatory effect of passive administration of allergen‐specific IgG4 antibodies in an animal model (*Gasser* et al., *unpublished data*). In this study, a mix of human Ara h 2‐specific mAbs increased safety and efficacy of OIT, reduced OIT‐induced IgE increases, and enhanced the production of endogenous allergen‐specific IgG in the mice. The latter suggests active involvement of IgG4 antibodies in the tolerization process.

Likewise, Burton et al. demonstrated in a food allergy model that administration of allergen‐specific IgG could prevent food allergy development, but also facilitated tolerance induction by increasing Treg responses and reducing Th2 and IgE responses when combined with oral immunotherapy.^135^ This process was shown to be mediated by FcγRIIB. These data indicate that allergen‐specific IgG4 antibodies can be used as standalone treatment, but may also be combined with AIT to increase safety and efficacy.

## 7 FURTHER POTENTIAL EFFECTS OF ALLERGEN‐SPECIFIC IgG AND IgA ANTIBODIES IN ALLERGY

### 7.1 Do allergen‐specific antibodies regulate the activation of and uptake across respiratory and intestinal epithelia?

As allergens can only affect the immune system when they become exposed to it, the transepithelial transport of allergens is crucial step of initiating allergic responses and symptoms. Intestinal as well as respiratory epithelia have been shown to express CD23, and its expression is upregulated in allergic disease.^136^, ^137^, ^138^ IgE is also present in the luminal side of the respiratory and gastrointestinal tract, and has been detected in saliva, feces, nasal fluid, and bronchoalveolar lavage fluid.^139^, ^140^, ^141^, ^142^ The concordant presence of the receptor on intestinal epithelium prompted studies to investigate a potential role of CD23 in transport of allergen‐IgE complexes.

Indeed several studies have shown that IgE‐allergen immune complexes are transported across the intestinal epithelial cells, and can thus promote the uptake of the allergens into the underlying tissues.^143^, ^144^, ^145^ Likewise, CD23 is also expressed on respiratory epithelium has been shown to play an important role in capturing and delivering allergens into the respiratory mucosa.^146^, ^147^ The high affinity receptor for IgE is also expressed on respiratory epithelial cells in asthma,^148^ and can reduce epithelial barrier function after crosslinking.^149^ However, FcεRI does not play a role in transporting IgE‐allergen immune complexes. This means that IgE and CD23 play an important role in the uptake of allergens across epithelia, thus promoting allergic inflammation.

In addition to CD23 two other (non‐classical) Fc receptors are present in intestinal and respiratory epithelia, the polymeric IgA receptor (pIgR) and the neonatal Fc receptor (FcRN). Both of these receptors specialize in the transport of immunoglobulins over epithelial sites. pIgR binds to the j‐chain that is needed to form dimeric IgA and pentameric IgM.^150^ Although pIgR expression is downregulated in asthma patients,^151^ pIgR is expressed in the nasal epithelium^152^ and in the lungs and respiratory tract where it localizes mainly to the secretory cells.^153^ In the gastrointestinal tract pIgR is expressed on epithelial cells.^154^ A recent publication has indicated that transport of antigen‐complexed IgA from the intestinal lumen into the tissues can also occur.^155^

Although it was described as a neonatal receptor transferring IgG across the placenta that is expressed in neonates, humans constitutively express FcrN on intestinal epithelia^156^ and bidirectional FcRN‐dependent transport of human IgG over epithelial cells has been shown in humans.^157^, ^158^, ^159^ Interestingly, FcRN is currently studied as a means of optimizing oral or nasal uptake of IgG.^160^, ^161^ FcRN is also expressed in the respiratory tract and can transport IgG antibodies from the lumen into the serosal tissue.^162^ Although it has not been studied to date, this transport via FcRN across respiratory epithelia may also be bidirectional, as it is in the intestine.

**FIGURE 4:**
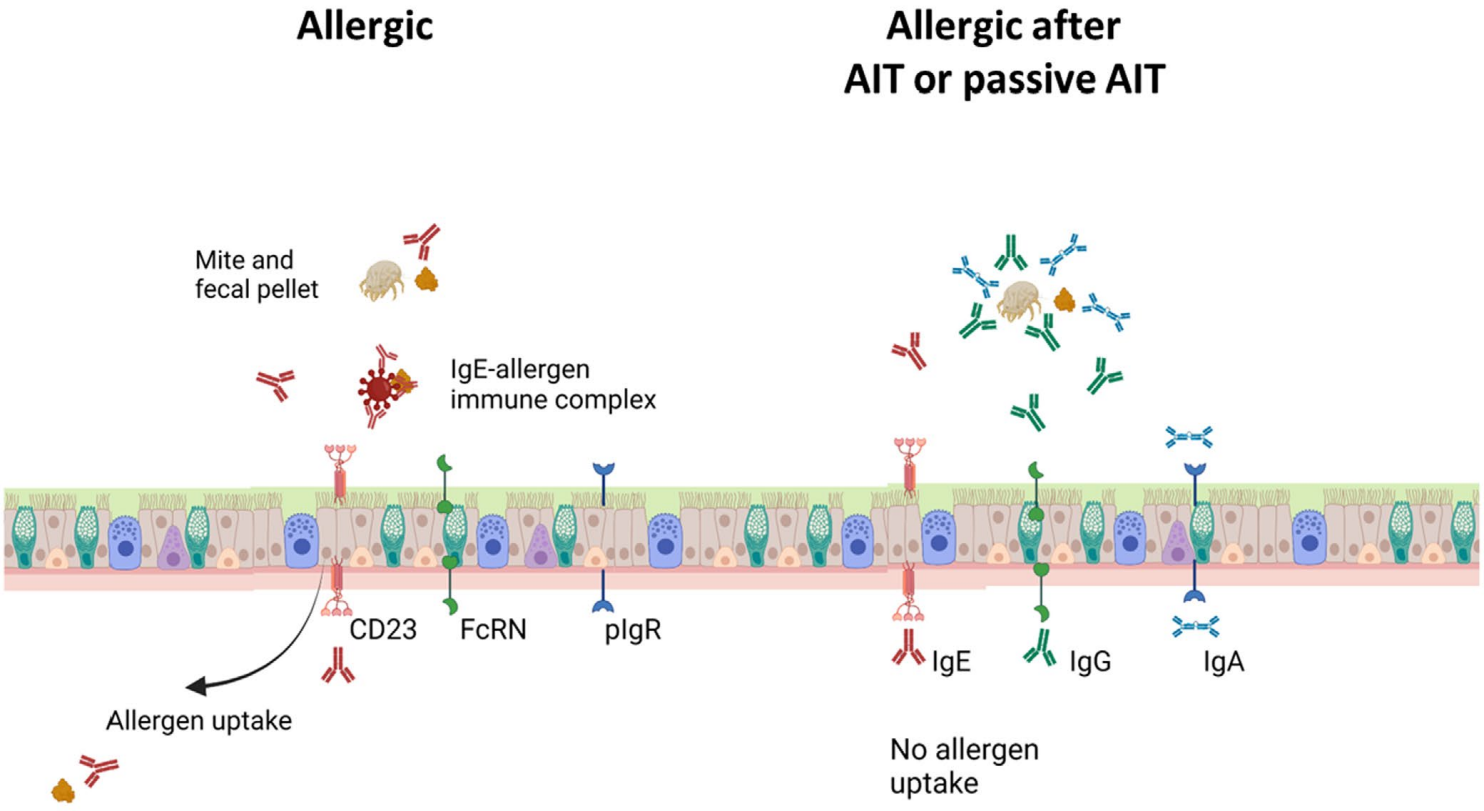
Hypothetical effect of allergen‐specific IgE, IgG, and IgA antibodies and allergen uptake over epithelia. Only allergen‐specific IgE, IgG, and IgA depicted. CD23 is expressed on respiratory and intestinal epithelium. It can pass the cell layer in both directions, and captures allergens in complex with IgE, that are transported into the underlying tissue. FcRN and pIgR that can transport IgG and dimeric IgA, transport IgG and IgA in two directions. However the fraction of specific IgG and IgA is very small and no allergen‐specific IgG and IgA antibodies are present on the luminal side. This changes after AIT when large amounts of allergen‐specific IgG and IgA antibodies are present and can prevent the formation of allergen‐IgE transport via CD23. As there is an excess of non‐specific IgG and IgA antibodies and the receptors bind monomeric IgA and IgG the allergen transfer across the epithelium is greatly reduced.

These findings indicate that both IgA and IgG can be actively transported over intestinal and respiratory epithelia. However, before AIT the fraction of allergen‐specific IgA and IgG is very low compared to the total levels, and very low levels of specific IgA and IgG can be expected there. After AIT or after passive AIT, allergen‐specific IgG and IgA can be deposited onto the epithelium, and prevent the CD23‐mediated uptake of allergen‐IgE complexes in the airways and intestines similar to preventing IgE‐allergen complex binding of these complexed to CD23 on B cells. This has not been studied yet, although it is clear that nasal fluid contains allergen‐specific antibodies that can inhibit IgE‐allergen binding to CD23 on B cells.^121^ Therefore, we hypothesize that allergen‐specific IgA or IgG (4) is deposited into the gastrointestinal as well as respiratory mucosa and can thus play a role in inhibiting allergen uptake into the body (Figure 4).

### 7.2 Does selective binding of allergen‐antibody immune complexes to different APC drive Th2 versus Treg and Th1/17 responses?

**FIGURE 5:**
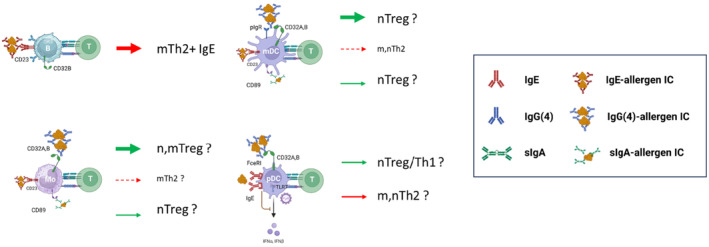
Hypothetical T cell differentiation biasing effects of selective targeting of allergen‐immune complexes to B cells, monocytes, mDC, and pDC via binding to different low‐affinity Fc receptors in part based on findings by Overman et al. (*unpublished data*). Depending on the isotype of the allergen‐specific antibody dominating before or after AIT, selective binding of the allergens will occur to B cells (IgE‐CD23 before AIT), monocytes and mDC (IgE‐CD23 before AIT but CD23 expression much lower than on B cells), monocytes (IgG(4)‐CD32A or IgA‐CD89 after AIT), mDC (IgG(4)‐CD32A or IgA‐CD89 after AIT) or pDC (IgG(4)‐CD32A or IgG‐FcRN after AIT). The size of the IC and thickness of the arrows indicates the relative contribution. The color of the arrows means that it is before AIT (red) or after AIT (green). Finally, the effect on activation of memory T cells (m) versus the activation and differentiation of naïve T cells (n) is indicated before the T cell type induced/activated.

It is now well established that different types of DC instruct naïve T cells to differentiate into Th2, Th1, Th17, Treg of Tfh cells.^163^ Selective targeting of allergens to different APC types could therefore lead to different CD4+ T cell polarization (Figure 5). Protein antigens can be taken up by fluid phase endocytosis in APC, but antigen presentation is much more efficient after binding to Fc receptors. Therefore, redirecting allergens to different APC may result in inducing a more Treg or Th1 phenotype in allergen‐specific cells.

It has been known since the 1980s that specific B cells are more efficient antigen presenting B cells than non‐specific B cells.^25^ Likewise antigen presentation induced by binding of allergen‐IgE complexes is 10–100 fold more efficient compared to fluid phase uptake and antigen presentation by B cells. However, when allergens are already bound to antibodies (neutralized by IgG or bound by IgE), allergen‐specific B cells may primarily need to use Fc receptor mediated antigen presentation as the B cell epitopes of the allergens are not available to the surface Ig of the specific B cells.

Immune complexes form when the ration between the antigen and the antibody is such, that both arms of the antibody bind to a different antigen, thus forming complexes of several antibodies and antigens.^164^ Similar to opsonized pathogens such complexes can then bind to Fc receptors. These are mainly expressed by antigen presenting cells. The high affinity receptors FcεRI, FcγRI (CD64), and FcαRI (CD89) are typically saturated by bound antibodies and bind uncomplexed antibodies. These are mainly expressed by antigen presenting cells. In contrast, low affinity receptors for IgG (CD32A, B, C and CD16), IgE (CD23) and the high affinity IgA receptor (CD89) have a low affinity for monomeric antibodies but a high affinity for complexed antibodies—in immune complexes or on opsonized microorganisms.

In an allergen‐specific IgE dominant environment—as in before AIT‐, the allergen will bind to IgE present on B cells, leading to antigen presentation to allergen‐specific T(h2) cells. As type‐2 precommitted IgG+ memory B cells express high levels and CD23^hi^, these cells are prone to switch to the production of IgE thus enabling long term IgE memory.^23^, ^24^

However, in an IgG (4) or IgA dominant environment—as in after AIT—IgE will be prevented from binding the allergen and thus to CD23+ B cells. This means that the allergen may be redirected to bind to monocytes, mDc, and pDC that express CD32 or CD89, and present the allergen to the T cells in a non Th2‐supportive context.

We hypothesize that different (iso) types of allergen‐specific antibodies target allergens to different APC types, leading to different patterns of CD4+ Th cell differentiation. Bheekha‐Escura et al. have suggested this already in 1995,^165^ but surprisingly this has never been investigated in detail afterwards. If this mechanism indeed plays a role, it means that the initial production of IgG after AIT plays an important role in the modulation of T‐cell activation and Treg induction as well.

Serum IgG can bind to three receptors on PBMCs. These receptors are FcγRI (CD64), FcγRII (CD32) and FcγRIII (CD16). CD64 is the high affinity IgG receptor that can bind monomeric IgG (1, 3 and 4).^166^ It is present on monocytes, dendritic cells, eosinophils and neutrophils, but in most cases the receptor is present only after induction, except on monocytes.^167^ CD32 is a low‐affinity receptor of which three isotypes exists, CD32a and CD32c that contain an intracellular ITAM motif, and CD32b that contains an ITIM motif (CD32b). Due to the low affinity of CD32 for monomeric IgG it is only capable of binding larger immune complexes.^168^

The isotypes of CD32 are differentially expressed on PBMCs with CD32a expressed on neutrophils, eosinophils, monocytes, macrophages, dendritic cells, platelets and basophils,^164^, ^169^ CD32b is expressed on B cells, neutrophils, eosinophils, monocytes, macrophages, dendritic cells and basophils,^164^, ^169^ and CD32c is only expressed by NK cells.^170^ The third IgG receptor CD16B is mainly expressed on neutrophils and CD16A on NK cells.^169^

Although IgG4 containing immune complexes are expected to bind to B cells that express high levels of CD32B, Gasser et al. recently noted that IgG4‐Bet v 1 immune complexes did not bind to B cells as expected but bound mainly to the monocytes and myeloid DC (Overman et al. *unpublished data*). Several studies have previously shown the ability of IgG4 to bind to CD32,^170^, ^171^, ^172^ and IgG4 has also been shown to bind to CD32B.^170^, ^171^ However the binding of IgG4 to CD32B is dependent on the size of the immune complex.^172^ In this referred study, immune complex binding of different IgG isoforms to TNP‐OVA was studied. The authors clearly demonstrated that the relatively small DNP‐OVA‐4 did not form IgG4 immune complexes capable of binding to CD32B, while the larger TNP‐OVA‐26 did. However, both types of IgG4 immune complexes could bind to monocytes (that express mainly CD32A).

Interestingly, normal IgG4 immune complexes are small in size.^128^ This is the because IgG4 in serum is functionally monovalent as a result of Fab arm exchange.^127^, ^129^ As B cells only express CD32B, it is of note that Bheekha‐Escura demonstrated that IgG immune complexes did not bind to B cells, unless the number of haptens in an IC was very high.^165^ As a result his means that allergens that are in complex with IgE bind to B cells via CD23, but that antigens bound by IgG4 do not bind to B cells. This may explain the findings by van Overman et al. (*unpublished data*).

Therefore, another role of IgG (4) antibodies in addition to blocking activity may be that they actively modulate immune responses via interaction with Fc receptors. We thus also hypothesize that immune complexes of IgG versus IgE allergen‐specific antibodies with allergens lead to differential binding to—and cytokine responses in—antigen presenting cells, leading to Treg versus Th2 differentiation. This may be the case by selective binding of allergens that are complexed to IgE versus IgG to distinct subtypes of antigen presenting cells. This selection of antigen presenting cell, can lead to differential APC activation, which in turn supports the development of allergen‐specific T cells into different cytokine producing patterns (Th2 vs. Treg vs. Th1 vs. Th17). If this mechanism indeed plays a role, it means that the initial production of IgG after AIT plays an important role in the modulation of T‐cell activation and Treg induction as well. As indicated in Figure 5, activation of memory Th2 cells before AIT is mainly induced by IgE‐allergen immune complexes binding to B cells. After AIT, binding of IgG‐allergen immune complexes is mainly to monocytes, mDC and pDC that activate memory Th2 cells and may bias this a little bit, but the amin effect will be through inducing new allergen‐specific T cells after activating naïve T cells that we presume will differentiate into Tregs (or Th1 cells), for example under the influence of IL‐10 induction in monocytes and mDC, and the restoration of IFN‐α production in pDC. Likewise, as CD89 crosslinking induces IL‐10, IgA‐allergen immune complexes may induce Tregs as well. However, this may not happen after subcutaneous AIT, but only after sublingual or oral AIT. After subcutaneous AIT IgA does not block IgE‐allergen immune complex binding,^26^ which indicates that the levels are too low to play a role similar to IgG (4).

### 7.3 Do AIT‐induced allergen‐specific IgG antibodies induce trained innate immunity?

A key development in our understanding of a broad memory in the innate immune system was the identification of innate immune training by the group of Netea in the Netherlands (See Box 1). Innate immune training is a mechanism that via epigenetic modification of DNA leads to the increased responsiveness of the innate immune system to TLR ligands, thus offering a broad innate protection against infection. Likewise, trained immune tolerance can develop, depending on the stimulus used during a primary exposure.

In addition, both the induction of innate immune training^173^ and innate immune tolerance by high dose LPS exposure in mattresses^174^ have been linked to increasing versus lowering the risk to develop allergic disease. As at high LPS exposure levels innate immune tolerance is induced, rather than training, this seems logical.^175^

Trained innate immunity in may be one of the mechanisms that influence the TLR‐mediated cytokine production in early life, and induces the epigenetic changes that are linked to the later development of food allergy and asthma (Box 1).

**FIGURE 6:**
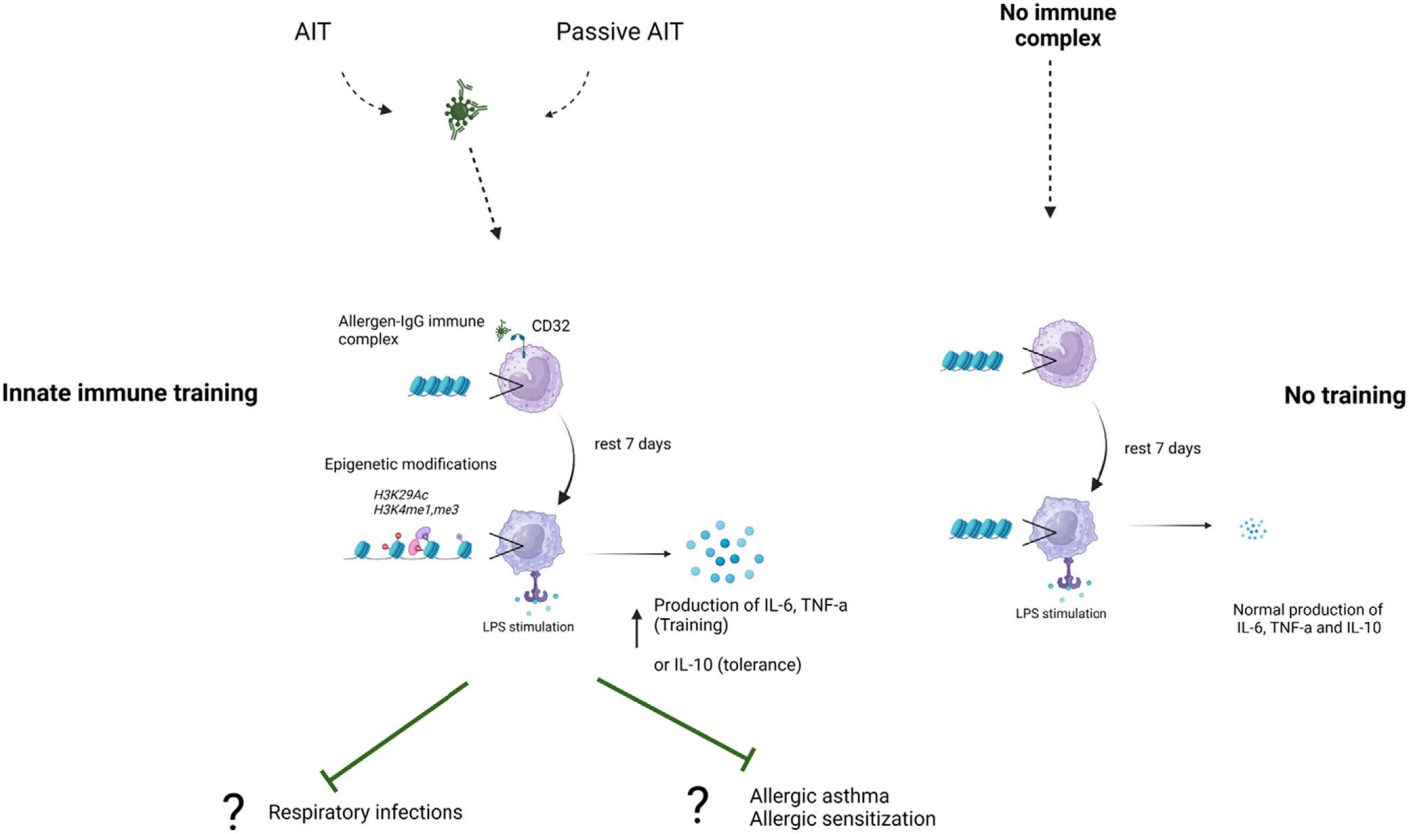
Hypothetical mechanism of innate immune training or tolerance by allergen‐IgG immune complexes. Bovine IgG aggregates and bovine IgG‐antigen immune complexes are known to bind to human monocytes via CD32 and induce innate immune training. Studies have only determined effects on IL‐6 and TNF‐α. We hypothesize that binding of allergen‐human IgG (4) immune complexes occurs to monocytes as shown by Overman et al. (*unpublished data*), and that similarly to bovine IgG, this leads to epigenetic changes that promote responses to TLR stimulation, resulting in increased cytokine production (IL‐6, TNF‐α: Training; IL‐10: Tolerance). As innate immune responses are crucial in the development of allergic responses, this may play a role in sensitization, but also in ongoing allergy. In addition, increase TLR responsiveness may reduce respiratory infections as well, which is relevant in asthma.

The term trained immunity has now been used for many epigenetics‐linked effects, and has also been used to explain the changes in ILC2 versus ILC1 and ILC3 number after AIT.^41^, ^110^ In addition to these findings we propose here that allergen‐specific IgG antibodies induced by AIT or administered in passive AIT may in fact induce trained innate immunity or ‐tolerance (Figure 6).

As discussed above, previous work in animal models has shown that IgG‐allergen immune complexes in maternal milk prevent allergic airway inflammation and induce immune tolerance towards the allergens.^65^, ^66^ These findings point to a more tolerogenic effect of IgG‐allergen immune complexes when given orally.

Given the notion that consumption of raw milk—that contains intact IgG—is inversely associated with allergic asthma and hay fever^71^ it is of interest that we demonstrated that bovine milk‐derived IgG could induce trained innate immunity.^176^, ^177^ In a follow up study using the RSV pre F protein, that is recognized by bovine IgG,^73^ we demonstrated that in fact IgG‐pre F protein immune complexes could induce trained immunity.^178^ These data indicated that immune complexes containing (bovine) IgG can induce innate immune training in human monocytes.

As bovine milk also contains IgG antibodies against respiratory allergens (Knol et al., *unpublished data*), it will be interesting to study if these immune complexes can also induce trained immunity of tolerance. At present it is not clear if *human* IgG‐containing immune complexes can form after AIT and have the same effect. However, as bovine IgG binds to human CD32,^73^ and allergen‐specific IgG4‐Bet v 1 immune complexes bind to human monocytes (*Overman* et al., *unpublished data*), AIT induced allergen‐specific IgG (4) may indeed train (or tolerize) the innate immune system.

## 8 CONCLUDING REMARKS AND FUTURE PERSPECTIVES

**FIGURE 7:**
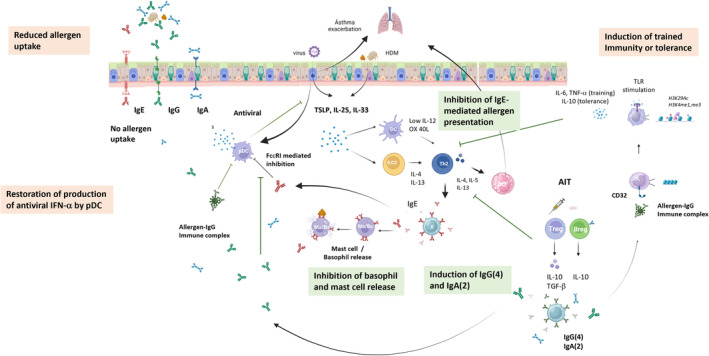
Allergen‐specific antibody mediated effects of AIT. In green boxes the known effect, in orange boxes the effects that we propose but that have not been proven yet. AIT induces allergen‐specific antibodies of the IgG (4) class. These are more prominent after subcutaneous AIT. IgA (2) is also induced, and these IgA antibodies are more prominent after sublingual/oral immunotherapy. These antibodies have a blocking function by neutralizing the allergen, and thus inhibiting basophil and mast cell release, but also IgE‐mediated allergen presentation to Th2 cells. A novel proposed mechanism is that these antibodies can also prevent the IgE‐mediated uptake of allergens across epithelia, prevent the IgE‐mediated blocking of antiviral IFN‐α responses by pDC, and can via interaction with FcγRII (CD32) induce trained innate immunity or tolerance.

Allergen‐specific IgE is the key antibody in allergy not only by mediating basophil and mast cell activation, but also by facilitating allergen presentation to Th2 cells. It is, however, of great relevance to consider allergen‐specific IgE in the context of other allergen‐specific antibody isotypes. The role of these other isotypes of allergen‐specific antibodies is becoming more and more clear (Figure 7).

Blocking activities of AIT‐induced and recombinant IgG antibodies are well known. Additional effects of these allergen‐specific IgG and IgA antibodies in oral tolerance induction to food and respiratory allergens, the role in regulating allergen uptake across epithelia, targeting allergens to selected antigen presenting cell types, restoration of antiviral pDC activity that is inhibited by IgE, and inducing trained immunity are just emerging and will be the focus of future research. In addition, the importance of allergen‐specific IgA on mucosal surfaces versus the role of IgG in systemic circulation and in the tissues remain to be established.

## AUTHOR CONTRIBUTIONS

Both authors conceived, wrote and edited the manuscript.

## CONFLICT OF INTEREST STATEMENT

Both authors have no conflict of interest.

BOX 1 *Trained innate immunity*

Trained immunity is a mechanism that explains how specific (pathogen‐derived) components can affect the secondary response of innate immune cells to Toll‐like receptor (TLR)‐triggering^179^, ^180^, ^181^, ^182^ This training effect is based on epigenetic changes in the trained monocytes/macrophages,^180^, ^181^ is long‐lasting and can still be noted after 3 months.^180^ In detail, they demonstrated that preincubation of peripheral blood mononuclear cell (PBMC)‐derived monocytes with the Bacille Calmette‐Guérin (BCG) vaccine significantly increased the response of the cells upon restimulation with a range of toll‐like receptor (TLR) ligands or heat‐killed bacteria. Most notably, this resulted in increased production of interleukin ILL‐6 and TNF‐α. Similar results were found upon training of innate immune cells within the PBMCs with the fungus C. albicans and C. albicans‐derived β‐glucan. This mechanism may be one of the key mechanisms that can explain the cross‐protection against non‐related pathogens, as this cannot be explained by simple cross reactivity of antibodies and T‐cells. Interestingly, BCG vaccination was recently shown to protect elderly people against respiratory tract infections of probable viral origin.^183^ All this work is summarized in two recent reviews.^184^, ^185^

*Epigenetics in Allergy*

Interestingly, epigenetic mechanisms have also been shown to be induced by respiratory viral infections,^186^ in environmental exposures such as the farming environment,^187^ and also by (raw) milk and early life nutrition components,^188^ as well as the microbial metabolite butyrate,^189^ all exposures that are linked to the development of allergic diseases. Indeed, multiple epigenetic changes have over the years been linked to immune function in allergic asthma as well as food allergy.^190^, ^191^, ^192^, ^193^

*Innate immune responses and allergy development*

Unfortunately limited data is available about the development of innate immune responses in early life and the link to the development of allergic diseases. In a key paper on innate immune development in early life Tulic et al demonstrated that innate cytokine production (IL‐6, TNF‐α, IL‐1β, and IL‐12) in response to TLR stimulation is significantly higher in cord blood of children that develop allergy (mostly respiratory) at the age 5 compared to non‐allergic children.^194^ The non‐allergic children however, had a lower cytokine response of these cytokines in stimulated in cord blood. At 5 years of age, the situation was reversed, with allergics producing lower levels of cytokines after TLR stimulation compared to the non‐allergics. This was previously also noted for the production of interleukin‐12 (IL‐12)^195^ Likewise, Halonen et al. described TNF‐a production (but not IL‐6, IL‐12 or IL‐10) in the first 3 months of life was increased in infants that would later develop asthma or eczema,^196^ and Zhang et al. reported that increased levels of IL‐6, IL‐1b, and TNF‐a were produced by cord blood of children that developed food allergies compared to non‐allergic children.^197^ When cultured in the presence of these cytokines, naïve Tregs downregulated Foxp 3 expression, and upregulated IL‐4, suggesting a role of the innate cytokine environment in regulating Th2/Treg development. Also in persistent food allergy, PBMC of 1 year old children In children with persistent egg allergy however, children of 1 year old with persistent egg allergy were shown to produce much higher levels of TNF‐α, IL‐6, IL‐1β Il‐8, and IL‐10.^198^ In contrast with these studies however. DeVries et al. recently described that neonatal trained immunity, based on the level of IL‐6 produced after TLR triggering of cord blood mononuclear cells, as well as by the presence of epigenetic modifications in cord blood, is protective against asthma development later in life.^199^

Interestingly, also maternal nutrition may affect the TLR‐responsiveness in infants after birth. Neonatal innate cytokine production responses to TLR stimulation were recently shown to be increased after maternal supplementation with vitamin D during pregnancy.^200^ Interestingly, in an animal model of allergic asthma, OM85—a polybacterial extract from a mixture of respiratory pathogens—when given during pregnancy was able to protect the offspring of the mice against allergic airway inflammation via epigenetic modifications.^201^ Together these data clearly indicate a link between the production of innate cytokines in response to TLR ligation in relation to allergy development and the possibility to modulate these responses in early life, potentially contributing to prevention of sensitization. Based on such findings, several recent reviews have discussed the potential role of trained immunity in allergy, including the potential of promoting trained immunity in preventing the development of‐ in allergy.^173^, ^202^, ^203^, ^204^, ^205^ However, in contrast the findings in early life, a recent study revealed an elevated trained innate immune phenotype in individuals with existing asthma.^206^ These data indicate that a trained innate immune phenotype may paradoxically be protective for development of allergic asthma induction but may have an inverse effect on existing asthma. Confusingly, it was recently shown that using the standard trained immunity protocol in newborn monocytes led to tolerization as evidenced by decreased TNF‐α, production upon stimulation, while adult monocytes were trained and produced increased TNF‐α levels.^207^ This could indicate that assessing the ability of components to induce training vs tolerance induction could depend on the age of the monocyte donor, suggesting that trained immunity picked up with adult monocytes may actually indicate tolerance induction of the component in early life.

The development of innate immune responses in early life is thus linked to the risk to develop allergies, and the timing of studying innate immunity is crucial for explaining these effects.

## Data Availability

Data sharing not applicable to this article as no datasets were generated or analysed during the current study.
